# Vapour-liquid-solid growth of ternary Bi_2_Se_2_Te nanowires

**DOI:** 10.1186/1556-276X-9-127

**Published:** 2014-03-18

**Authors:** Piet Schönherr, Liam J Collins-McIntyre, ShiLei Zhang, Patryk Kusch, Stephanie Reich, Terence Giles, Dominik Daisenberger, Dharmalingam Prabhakaran, Thorsten Hesjedal

**Affiliations:** 1 Clarendon Laboratory, Department of Physics, University of Oxford, Parks Road, Oxford OX1 3PU, UK; 2 Fachbereich Physik, Freie Universität Berlin, Arnimallee 14, Berlin 14195, Germany; 3 Diamond Light Source, Chilton, Didcot OX11 0DE, UK

**Keywords:** Nanowires, Topological insulators, VLS growth, Raman spectroscopy

## Abstract

High-density growth of single-crystalline Bi_2_Se_2_Te nanowires was achieved via the vapour-liquid-solid process. The stoichiometry of samples grown at various substrate temperatures is precisely determined based on energy-dispersive X-ray spectroscopy, X-ray diffraction, and Raman spectroscopy on individual nanowires. We discuss the growth mechanism and present insights into the catalyst-precursor interaction.

## Background

Topological insulators (TIs) are characterised by insulating behaviour in the bulk and counter-propagating, spin-momentum-locked electronic surface states that are protected from backscattering off nonmagnetic impurities by time-reversal symmetry [1-7]. It is an experimental challenge to measure the topological surface states in electrical transport experiments, as defect-induced bulk carriers are the main contribution to the measured conductance [8]. In principle, there are two ways to overcome this problem. First, materials engineering can be employed; this allows for compensation doping or reduction of the intrinsic defects [9-11]. Examples are Bi_2_Te_2_Se (BTS) and Bi_2_Se_2_Te (BST) - a combination of the binary TIs Bi_2_Se_3_ and Bi_2_Te_3_ with tetradymite structure [12]. These ternary compounds have a higher bulk resistivity due to suppression of vacancies and anti-site defects [13]. Accordingly, BST was recently found to have dominant surface transport properties [14].

The second approach is to reduce the crystal volume with respect to the surface area. Nanostructures such as thin films or nanowires have high surface-to-volume ratios, enhancing the contribution of surface states to the overall conduction [15,16]. Signatures of surface effects are readily observed in Bi_2_Se_3_ nanoribbons, but n-type doping due to Se vacancies is identified as a major obstacle for TI-based devices [16,17].

Here we report the growth of BST nanowires- a promising combination of optimised materials composition and nanostructures. So far, the high-purity growth of uniform TI nanowires has not been achieved through the vapour-liquid-solid (VLS) method [18,19]. We present a detailed study of sample growth as a function of substrate temperature using scanning electron microscopy (SEM), transmission electron microscopy (TEM), energy-dispersive X-ray spectroscopy (EDS), X-ray powder diffraction, Raman spectroscopy, and atomic force microscopy (AFM).

## Methods

The samples were grown employing an Au-assisted VLS process. Si(100) substrates were functionalised with 0.1% poly-L-lysine solution (PLL) and coated with colloidal 5-nm-diameter Au nanoparticles. A solid precursor was placed in the centre of a Nabertherm B180 horizontal tube furnace (Lilienthal, Germany) at atmospheric pressure and at a constant N_2_ flow rate of 150 standard cubic centimetres (sccm). Prior to growth, the tube was flushed several times by pumping with a membrane pump and readmitting dry nitrogen. The furnace was ramped to the desired temperature over 1 h and then held constant for 1 h, before being allowed to cool down to room temperature. The substrates were placed downstream from the precursor. By adjusting the position, substrate temperatures between 150°C and 550°C can be set for a chosen centre temperature of 585°C.

SEM and EDS measurements were carried out on as-grown samples. For TEM measurements, nanowires were scraped from the substrate and placed onto a carbon support film on a copper grid. For tapping-mode AFM measurements, the nanowires were transferred onto a clean Si substrate in a frozen drop of DI water.

X-ray powder diffraction data were measured on beamline I15 at the Diamond Light Source in Didcot, Oxfordshire, England. A pre-focused monochromatic beam (*E*=37.06 keV) was collimated with a 30 - *μ*m pinhole. The sample material was removed from the as-grown substrate using a micro-chisel and placed onto the culet of a single crystal diamond (as used in diamond anvil cell experiments). In this way, diffraction patterns free of substrate contributions can be recorded. At these energies, there is little absorption by diamond and the diamond background scattering and Bragg contributions are easily identified. Powder diffraction patterns were recorded using a PerkinElmer detector (Waltham, MA, USA), integrated using Fit-2D and analysed using PowderCell.

Raman spectroscopy was carried out on a Horiba T64000 Raman spectrometer system (Kyoto, Japan) in combination with a 632.8 -nm He-Ne laser at 1 mW. The beam was focussed onto the substrate through a microscope with a ×100 objective lens to allow for the study of individual nanowires. The backscattered signal was dispersed by a triple grating spectrometer with a spectral resolution of 1 cm ^−1^. The polarisation of the light was parallel to the nanowire axis to maximise the intensity. All measurements were carried out at room temperature. The spectrometer was calibrated using a Ne standard.

## Results and discussion

**Figure 1:**
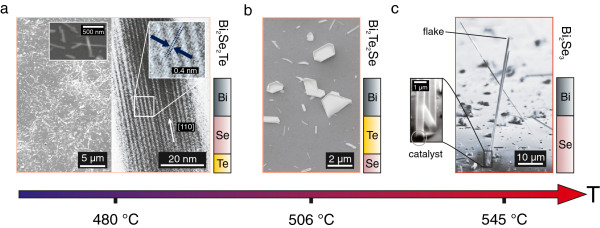
**Electron micrographs of samples grown at various temperatures and their composition.(a)** 480°C (left: 45° tilt-view SEM, right: TEM), **(b)** 506°C (top-view SEM), and **(c)** 545°C (side-view SEM). In the lattice-resolved TEM micrograph in **(a)**, the indicated growth direction is along [110]. The inset reveals an interplanar distance of 0.4 nm. In **(b)**, mostly Bi_2_Te_2_Se platelets are observed, whereas at higher temperatures **(c)**, the sample is composed of flakes as well as large Bi_2_Se_3_ wires.

The morphology and composition of the synthesised nanostructures depend strongly on the substrate temperature. SEM micrographs of samples grown at substrate temperatures of 480°C, 506°C, and 545°C are shown in Figure 1 together with the composition of the grown structures. Details about the determination of the composition are given below. At the highest temperature (see Figure 1c), mostly small objects were found and in part sheets were growing out of the surface, along with sparsely distributed larger wires. The composition is Bi_2_Se_3_, indicating that the temperature is too high for the incorporation of Te.

**Figure 2:**
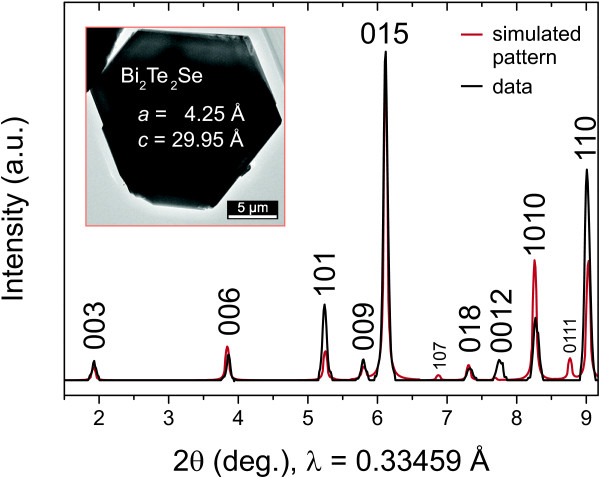
**X-ray powder diffraction pattern of the nanostructure sample grown at 506 *° *C (black line).** The pattern is assigned to Bi_2_Te_2_Se. The underlying red trace is the simulated pattern [20]. The inset shows a TEM micrograph of a hexagonal platelet, which is typical for the studied powder sample.

At 506°C, the planar growth increases and only a few, smaller nanowires are found as shown in Figure 1b. The X-ray powder diffraction pattern of a powder obtained from the as-grown material by scraping (cf. Figure 2) shows that the material is BTS with space group $R\bar{3}m$ and the lattice parameters *a*=4.25 Å and *c*=29.95 Å [20]. The peak associated with [110]-oriented crystals is enhanced, suggesting a preferred orientation within the sample. For two peaks, (107) and (01.11), the intensity is too low to be resolved.

At a substrate temperature of 480°C, the surface is uniformly covered with nanowires, indicating that the axial growth dominates over the planar and radial growth modes as can be seen in Figure 1a. TEM-based EDS analysis identifies the composition as BST. Lattice-resolved TEM imaging shows a spacing of 0.4 nm between adjacent lattice planes, consistent with a growth direction along [110]. This confirms the observation of a preferred growth orientation in the X-ray data of the sample grown at 506°C.

At even lower temperatures, i.e. below the optimum BST growth temperature (results not shown), axial and radial nanowire growth still dominates. These nanowires contain no Bi, since its vapour pressure is orders of magnitude lower than that of Se and Te at these temperatures.

**Figure 3:**
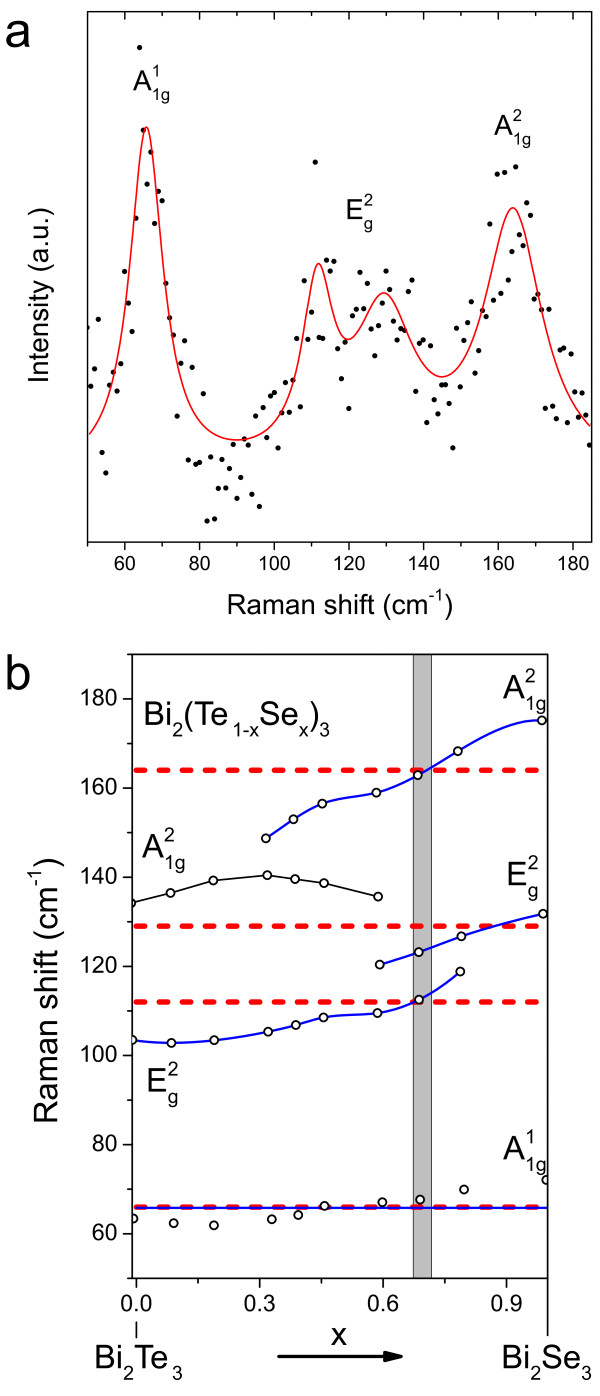
**Raman spectrum of a single nanowire and representation of the Raman data for Bi**_**2**_**(Te **_1−***x***_**Se**_***x***_**)**_**3**_**.(a)** Raman spectrum of a nanowire grown at 480°C. Four peaks at 66, 112, 129, and 164 cm ^−1^ are obtained from fitting Lorentzians. The peaks can be assigned to the Raman modes of Bi_2_Se_2_Te. **(b)** Representation of the Raman data for Bi_2_(Te _1−*x*_Se_*x*_)_3_ for 0<*x*<1 taken from Richter and Becker [21]. Data points of the higher A${\;}_{1g}^{2}$ and the E${\;}_{g}^{2}$ mode were fitted with a spline interpolation. Red dashed lines indicate the peak positions of the nanowire and the shaded grey area the composition range of *x*≈0.69±0.02.

The composition of the nanostructures is further analysed using micro-Raman spectroscopy, which allows for a more precise study of the nanowires than EDS without the need of a large amount of sample material. The spectrum of a single nanowire grown at 480°C is shown in Figure 3a and exhibits four peaks that were assigned to the three modes of BST - note that the $E_{g}^{2}$ mode is split for certain stoichiometries. The Raman spectrum of Bi_2_(Te _1−*x*_Se_*x*_)_3_ strongly depends on the compositional value *x*, as determined by Richter and Becker (data reproduced in Figure 3b) [21]. The spectrum is dominated by the A${\;}_{1g}^{2}$ and E${\;}_{g}^{2}$ modes over a broad range of intermediate *x* values due to the decoupling of the Bi-Te and Bi-Se vibrations at high frequencies. The A${\;}_{1g}^{2}$ mode especially is subject to change for high Se concentrations. This fact makes this mode a sensitive indicator of variations in the concentration *x*. The high-frequency E${\;}_{g}^{2}$ mode is broadened as in the original data of Richter and Becker [cf. their Figure five(a)]. The position of the A${\;}_{1g}^{2}$ and the higher E${\;}_{g}^{2}$ mode was weighted stronger than the position of the relatively constant A${\;}_{1g}^{1}$ mode and the lower E${\;}_{g}^{2}$ mode. The value of *x* was determined to be 0.7, corresponding to BST.

**Figure 4:**
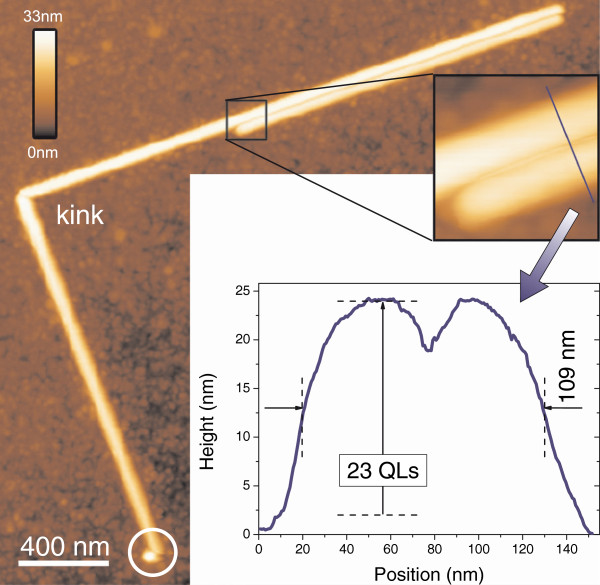
**AFM micrographs of Bi**_***2***_**Se**_***2***_**Te nanowires on Si.** Two nanowires are visible which stick together side by side, having a diameter (height) of 22.0 nm or 23 quintuple layers (QLs).

Figure 4 shows an AFM scan of two nanowires with a length between 1.5 and 5 *μ*m and a width of 54.5 nm (full width at half maximum). The diameter (measured height) of the nanowires is 22.0 nm, corresponding to 23 quintuple layers (QLs) with 1 QL = 0.96 nm. We can conclude that these nanowires were grown along the [110] direction.

The VLS growth mechanism requires the formation of a catalyst-precursor alloy and the subsequent crystallisation out of the supersaturated solution [22]. A metal alloy particle is typically either found at the tip or the root of the nanowire [23]. The samples show root-catalysed growth as can be seen in Figure 1c. A catalyst particle is found at the base of all of the nanowires investigated at this temperature.

Tip-based Bi_2_Se_3_ nanowire growth was observed by Kong et al. using 20-nm-diameter Au particles in an identical experiment [24]. In contrast, Alegria et al. reported root-based growth of Bi_2_Se_3_ nanostructures from an annealed, 5-nm-thick Au layer using metal-organic chemical vapour deposition [18]. The differing growth mechanism was explained by the use of a gas source instead of a solid precursor. Our study suggests that it is not the growth technique that determines the VLS growth mechanism, but rather the size of the catalytic particle. Above a critical size, the catalytic particle is lifted up by the growing nanowire as observed by Kong et al. This effect can be explained by a catalyst-substrate interaction that depends on the size of the catalyst particle. If the Au catalyst alloys with the SiO_2_/Si substrate, e.g. driven by size-dependent melting point reduction, it will not be lifted up by the growing wire but stay at the interface with the substrate. For larger catalyst particles, alloying is still expected at the boundary of the particle, but the overall anchoring to the substrate is too weak and the particle is lifted up as the wire grows.

**Figure 5:**
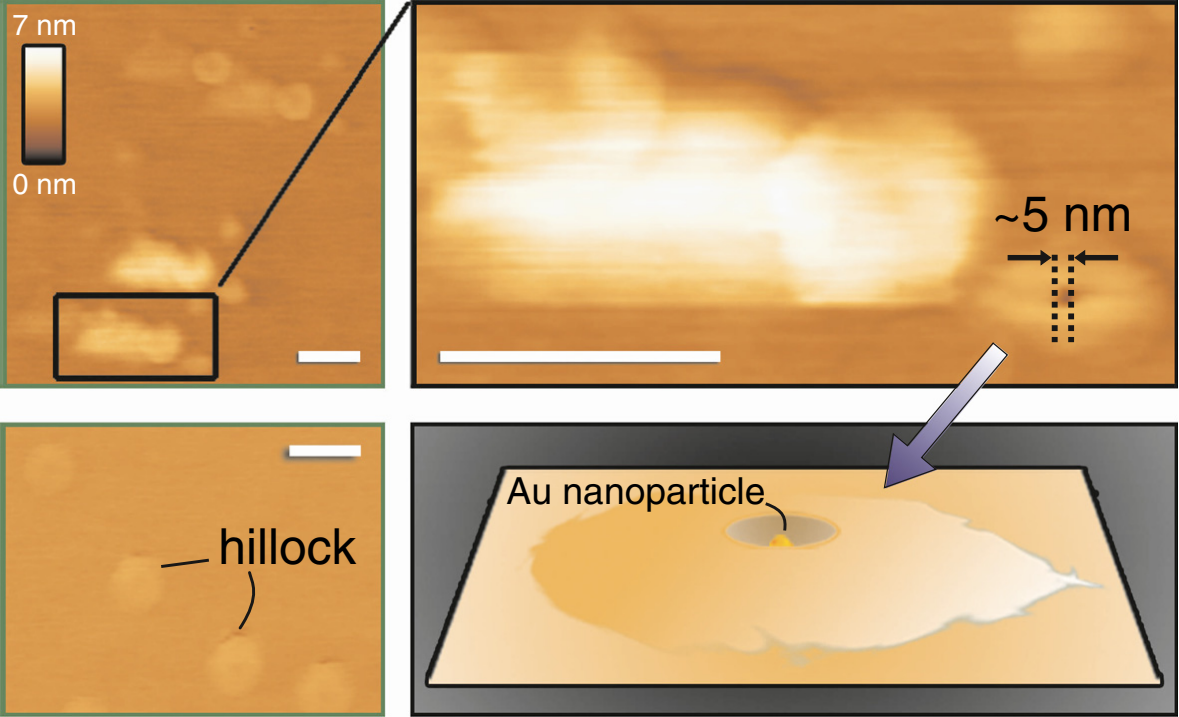
**AFM images of Au catalyst and deposited precursor material at early stage of VLS growth.** The catalyst-precursor mounds are indicated in the image. The scale bars correspond to 100 nm.

The AFM investigation of a sample removed at an early stage of the growth process gives further insight into the working of the catalyst particle. AFM scans reveal rounded mounds with an indentation in their centre as shown in Figure 5. The width of the structure in the centre of the indentation is 5 nm - the same as the diameter of the Au catalyst particles. This material has no apparent structure and does not show any symmetry or characteristic QL-high steps. Structures with a similar shape were reported to appear in studies of SiO_2_ encapsulation of Au nanoparticles on Si substrates upon annealing in oxygen atmosphere [25]. The observed mounds are too small to identify the composition unambiguously using EDS. It is unlikely that they are SiO_2_, since our experiments were carried out under N_2_ atmosphere. If the unspecified material is the precursor, it gives evidence of an early stage of the alloy particle. Firstly, the Au particle does not facilitate a permanent metal precursor formation. Secondly, Au particles merely provide nucleation centres that promote precursor deposition but are subsequently buried. This agrees with the possibility of catalyst-free synthesis of Bi_2_Se_3_ nanostructures [26].

## Conclusions

In summary, we present the VLS growth of stoichiometric Bi_2_Se_2_Te (BST) nanowires. A comparison of growth at different substrate temperatures reveals its strong influence on the morphology and composition of the nanostructures. High-density BST nanowire growth only occurs at 480°C, as determined by SEM EDS and Raman spectroscopy. The nanowires grow as single crystals along [110] with diameters of ≈55 nm. At a slightly higher temperature (506°C), the composition and morphology change to Bi_2_Te_2_Se nanostructures. They display high phase purity in powder X-ray diffraction experiments. The analysis of the growth mechanism has shown that Au nanoparticles rest at the root of the nanowire facilitating root-catalysed VLS growth. This growth mode is in contrast to the tip-catalysed growth of Bi_2_Se_3_ nanowires and nanoribbons using larger Au nanoparticles [24]. Our findings give new insight into the formation of the catalyst-precursor alloy and the nanoparticles acting as nucleation centres for the growth of ternary chalcogenide nanowires. This work represents an important step towards functionalising TI nanowires for spintronic devices.

## Competing interests

The authors declare that they have no competing interests.

## Authors’ contributions

PS and TH conceived the study. PS carried out the CVD growth with the help of SZ and was involved in all characterisation experiments. DP grew the bulk samples. PK and SR carried out the Raman studies, and TG and DD the XRD studies. LCM was responsible for the XRD analysis. TH performed the AFM studies and wrote the manuscript. All authors read and approved the final version of the manuscript.
